# Enhanced Biomass and Astaxanthin Production of *Haematococcus pluvialis* by a Cell Transformation Strategy with Optimized Initial Biomass Density

**DOI:** 10.3390/md18070341

**Published:** 2020-06-29

**Authors:** Feng Li, Minggang Cai, Mingwei Lin, Xianghu Huang, Jun Wang, Hongwei Ke, Chunhui Wang, Xuehong Zheng, Ding Chen, Shihui Yang

**Affiliations:** 1 College of Fisheries, Guangdong Ocean University, Zhanjiang 524088, China; lifeng2318@gdou.edu.cn (F.L.); huangxh@gdou.edu.cn (X.H.); 2 Fujian Provincial Key Laboratory for Coastal Ecology and Environmental Studies, Xiamen University, Xiamen 361102, China; 3 Key Laboratory of Marine Chemistry and Applied Technology, Xiamen 361101, China; Hongwei_KE@xmu.edu.cn (H.K.); springfl@xmu.edu.cn (C.W.); xhzheng@xmu.edu.cn (X.Z.); chending@xmu.edu.cn (D.C.); 4 College of Ocean and Earth Science, Xiamen University, Xiamen 361101, China; 22320142200997@stu.xmu.edu.cn (M.L.); wangjun@mail.maritech.com.cn (J.W.); 22320191150991@stu.xmu.edu.cn (S.Y.); 5 Department of Biotechnology, Xiamen Ocean Vocational College, Xiamen 361101, China

**Keywords:** *Haematococcus pluvialis*, astaxanthin, encystment, cell transformation, initial biomass density

## Abstract

Astaxanthin from *H. pluvialis* is an antioxidant and presents a promising application in medicine for human health. The two-stage strategy has been widely adopted to produce astaxanthin by the *Haematococcus* industry and research community. However, cell death and low astaxanthin productivity have seriously affected the stability of astaxanthin production. This study aims to test the effect of cell transformation strategies on the production of astaxanthin from *H. pluvialis* and determine the optimal initial biomass density (IBD) in the red stage. The experimental design is divided into two parts, one is the vegetative growth experiment and the other is the stress experiment. The results indicated that: (1) the cell transformation strategy of *H. pluvialis* can effectively reduce cell death occurred in the red stage and significantly increase the biomass and astaxanthin production. (2) Compared with the control group, the cell mortality rate of the red stage in the treatment group was reduced by up to 81.6%, and the biomass and astaxanthin production was increased by 1.63 times and 2.1 times, respectively. (3) The optimal IBD was determined to be 0.5, and the highest astaxanthin content can reach 38.02 ± 2.40 mg·g^−1^. Thus, this work sought to give useful information that will lead to an improved understanding of the cost-effective method of cultivation of *H. pluvialis* for natural astaxanthin. This will be profitable for algal and medicine industry players.

## 1. Introduction

Oxidative stress is a negative effect produced by free radicals in the body, which can lead to an altered intracellular redox status causing cellular dysfunction or death, and the pathogenesis of numerous diseases is associated with it [1,2]. Natural antioxidants help counteract the negative effects of oxidative stress and other related factors, minimizing the influence of oxidative damaging processes [2,3]. As a red ketocarotenoid with powerful biological antioxidant activity [4], natural astaxanthin has considerable potential and promising applications in medicine for human health [5,6,7]. Astaxanthin can be synthesized by several microorganisms in the marine environment [8,9]. In these reported sources, the green microalgae *Haematococcus pluvialis* has emerged as a promising cell factory for natural astaxanthin since it has the highest capacity to accumulate astaxanthin [10,11].

*H. pluvialis* evolved various strategies for defense mechanisms to cope with environmental stress, such as encystment formation and astaxanthin biosynthesis [12,13]. When the surrounding environmental conditions gradually became unfavorable from favorable, environmental stress firstly induced *H. pluvialis* cell encystment to make cell morphological changes from motile cells to nonmotile cells [14,15]. With prolonged stress, cells further developed into red cysts accompanied by astaxanthin accumulation [16,17]. In the natural environment, this cell transformation process was carried out slowly, so rarely observed the massive death of cells. However, in the actual production of *H. pluvialis* with the two-stage approach as the main strategy, considerable cell death due to the environment changed suddenly after the vegetative cells transferred into the red stage from the green stage [18,19,20] and this has seriously affected the astaxanthin production. Thus, searching for an advanced culture strategy of *H. pluvialis* is critical to achieving the efficient and stable production of astaxanthin.

The previous study suggested that those strains with predominant nonmotile cells were more suitable for astaxanthin production in the red stage than those appearing as motile cells [21,22,23,24]. The division rate of the motile cell was significantly higher than that of the nonmotile cell [25], so it was more suitable for the production of biomass in the green stage. We believe that the rational use of the biological characteristics of different types of *H. pluvialis* cells can help to improve the production of astaxanthin. For this reason, we propose an efficient method for producing astaxanthin using the cell transformation strategy as follows: (1) cultivate the motile cells to produce more cells, (2) collect these cells and induct them encystment to form the nonmotile cells, and (3) induce these nonmotile cells to accumulate astaxanthin. In this work, we conducted comparative experiments between two different strategies for the production of astaxanthin to assess the biomass and astaxanthin production of *H. pluvialis* in a cell transformation strategy and to determine the optimal initial biomass density (IBD) in the red stage. 

## 2. Results

### 2.1. The Cell Morphology and Growth in the Vegetative Growth Stage

**Figure 1:**
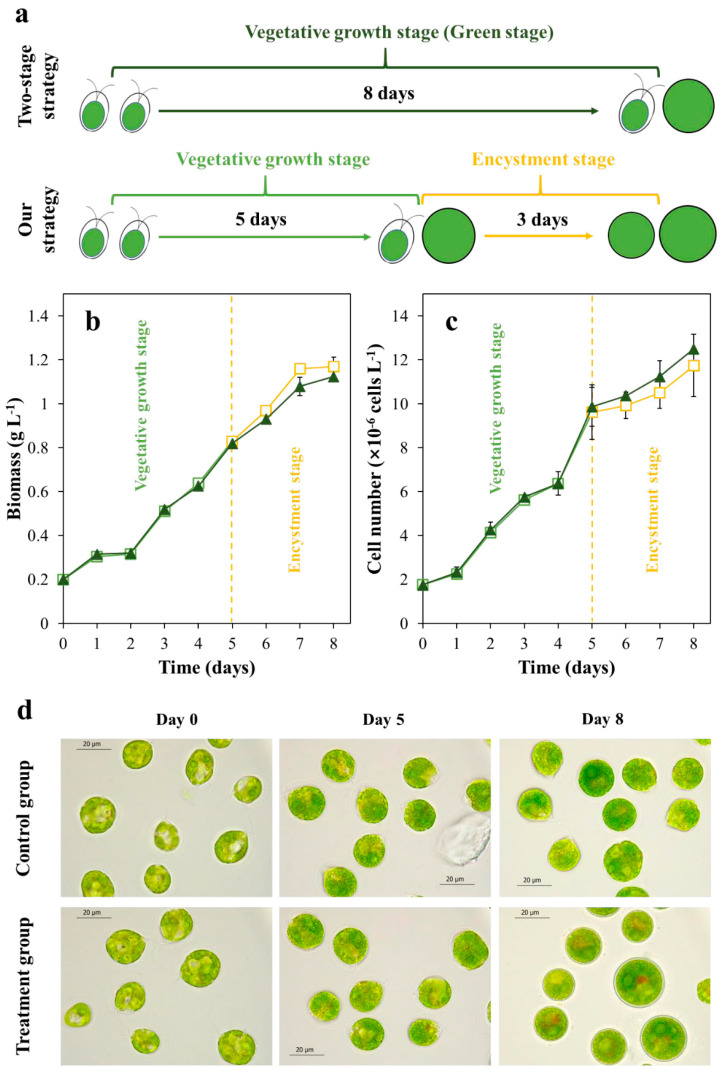
The experimental design of the vegetative growth stage (**a**). The biomass (**b**), cell number (**c**), and cell morphological changes (**d**) of two groups in the vegetative growth stage (Dark green triangle: the data in the control group. Green square: the data of vegetative growth stage in the treatment group. Orange square: the data of encystment stage in the treatment group).

The experimental design of the vegetative growth stage is shown in Figure 1a. In the two-stage strategy (control group), motile cells were cultured under favorable conditions for eight days (vegetative growth stage, also called green stage). In our strategy (treatment group), motile cells are first cultured under favorable conditions for five days (vegetative growth stage), then transferred into phosphorus-free condition containing 1g·L^−1^ NaCl to treatment for three days (encystment stage). 

We firstly investigated the changes in cell growth of two groups. No difference in the biomass (Figure 1b) and cell number (Figure 1c) was observed between the control and treatment groups during the first five days. After that, it was observed that the biomass of the treatment group increased slightly compared with the control group, but the cell number was lower than that of the control group. 

Changes of cell morphology were observed under the light microscope, and the results are shown in Figure 1d. The motile cells were used as “inoculum” for experiments (day 0). After five days of cultivation, a few nonmotile cells appeared in both cultures. At the end of the eight-day culture, the cells in the control group are mainly vegetative cells, which are a mixture of motile cells and nonmotile cells. The vegetative cells in the treatment group due to transfer into the encystment environment on day 5, so at the end of the culture, almost all the cells were green nonmotile cells. 

### 2.2. The Biomass, Astaxanthin Concentration, and Cell Morphology in the Red Stage

**Figure 2:**
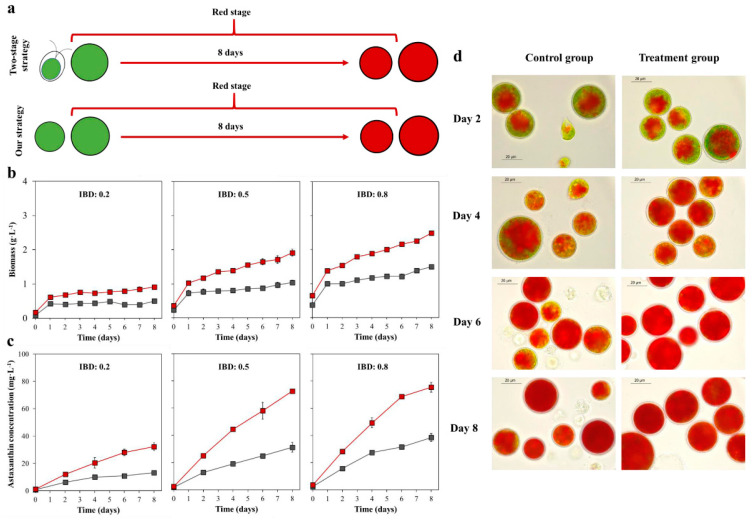
The experimental design of the red stage (**a**). The biomass (**b**) and astaxanthin concentration (**c**) of the control group (Gray) and treatment group (Red) under three IBDs in the red stage. The cell morphological changes (**d**) of two groups under 0.5 IBD.

The experimental design in the red stage was shown in Figure 2a. The cells at the end of the previous experiment were collected (vegetative cells in the control group and green nonmotile cells in the treatment group), then transferred into stress conditions at 0.2, 0.5, and 0.8 IBD for eight days of induction. The first specific objective was to determine the differences in astaxanthin accumulation between vegetative cells and nonmotile cells. A second objective was to assess the effect of IBDs on the production of astaxanthin.

As shown in Figure 2b, all the biomass of 0.2, 0.5, and 0.8 IBD groups demonstrated a rapid increase on the first day followed by a steady rise until the end of induction. The maximum biomass of the treatment group in three IBD groups was higher than that of the control group. As shown in Table 1, the maximum biomass of treatment group respectively reached 0.92 ± 0.06, 2.02 ± 0.03, and 2.62 ± 0.06 g L^−1^ in 0.2, 0.5, and 0.8 IBD group, which was about 1.59, 1.63, and 1.58 times as high as that of the control group. The maximum biomass productivity of 0.44 ± 0.05, 0.66 ± 0.04, and 0.72 ± 0.05 g L^−1^ d^−1^ was obtained in the treatment group, which was about 0.29, 0.32, and 0.16 times respectively higher than that of the control group in 0.2, 0.5, and 0.8 IBD groups.

As shown in Figure 2c, the astaxanthin concentrations in both groups increased with time. A significant difference in astaxanthin concentration was observed between the control and treatment groups. The highest astaxanthin concentration of the treatment group in 0.2, 0.5, and 0.8 IBD group reached 32.26 ± 2.76, 72.51 ± 0.82, and 75.53 ± 3.55 mg L^−1^ (Table 1), which was 2.07, 2.09, and 1.73 times as high as that of the control group, respectively. The maximum astaxanthin productivity of 5.42 ± 0.59, 11.36 ± 0.83, and 12.37 ± 0.02 mg L^−1^ d^−1^ was obtained in the treatment group, which was about 0.96, 1.10, and 0.85 times respectively higher than that of the control group in 0.2, 0.5, and 0.8 IBD group.

Figure 2d shows the cell morphology changes of two groups during the red stage. Only 0.5 IBD group were shown here. After two days of induction, red pigmentation began to appear towards the center of the cell. A large number of motile cells with red pigment can still be observed in the control group, but not in the treatment group. As the stress persisted, the red color of the cells in the two cultures deepened further, eventually forming the red cysts. Further, more cell death was observed in the control group than in the treatment group, especially in the late induction period.

### 2.3. The Astaxanthin Content and Astaxanthin Content Increase Rate in the Red Stage

**Figure 3:**
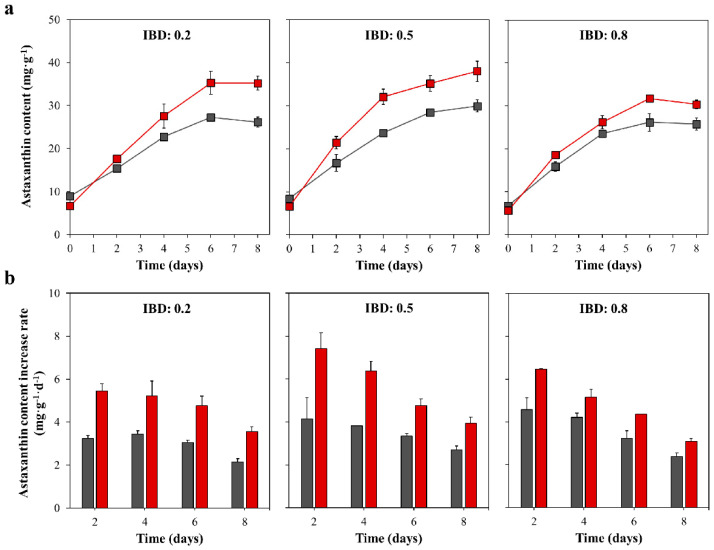
The astaxanthin content (**a**) and astaxanthin content increase rate (**b**) of control group (Gray) and treatment group (Red) under different IBDs in the red stage.

The data of astaxanthin content in Figure 3a shows that the highest astaxanthin content in the treatment group occurred in a 0.5 IBD group, followed by 0.2 and 0.8 IBD groups. In the 0.5 IBD group, the maximum astaxanthin content of the treatment group reached 38.02 ± 2.40 mg g^−1^ after 8 days of induction (Table 1), which was ca. 7.7% and 14.0% higher than that in 0.2 and 0.8 IBD group. From the comparative analysis of the control group and the treatment group, the maximum astaxanthin content of the treatment group in 0.2, 0.5, and 0.8 IBD groups was 29.3%, 26.8%, and 27.3% higher than that of the control group, respectively. 

To further assess the ability of astaxanthin accumulation between vegetative- and nonmotile cells, the astaxanthin content increase rates were calculated. As shown in Figure 3b, the cells in the treatment group showed a stronger astaxanthin accumulation ability than the control group. From Table 1, the maximum astaxanthin content increase rate of the treatment group in 0.2, 0.5, and 0.8 IBD group reached 0.54 ± 0.03, 0.74 ± 0.07, and 0.65 ± 0.00 mg g^−1^ d^−1^, which was about 1.59, 1.80, and 1.41 times as high as that of the control group, respectively. 

The cell mortality rate of all cultures was tested at the end of induction. According to Table 1, the cell mortality rate of the control group and the treatment group showed a similar trend, that is, the 0.2 IBD group had the highest cell mortality rate, followed by the 0.5 and 0.8 IBD groups. In the same IBD group, the treatment group had a lower cell mortality rate than the control group. The cell mortality rate in the treatment group in 0.2, 0.5, and 0.8 IBD group reached 7.63 ± 3.35%, 4.56 ± 0.27%, and 3.51 ± 2.48%, which were reduced by about 70.5%, 80.3%, and 81.6% compared with the control group, respectively.

## 3. Discussion

The culture conditions for maximum growth and maximum astaxanthin content in the *H. pluvialis* cultivation are mutually exclusive [26]. This is because the rapid growth of *H. pluvialis* requires favorable conditions [27,28], and the accumulation of astaxanthin requires stress conditions [29,30,31]. Although the two-stage strategy has solved the conflict between growth conditions and astaxanthin accumulation conditions well, the problem of massive cell death occurred in the red stage has not been effectively resolved, which is the main reason for the low overall productivity of astaxanthin. In this study, the cell mortality rate of the treatment group in the red stage was significantly lower than that of the control group. Since the initial cultures in the treatment group were almost all nonmotile cells with thick cell walls, and the initial cultures of the control group contained both nonmotile cells and motile cells, we believe that the high cell mortality rate in the red stage of the two-stage strategy may be attributed to the presence of a large number of motile cells in the culture. This is supported by previous researches that the motile cells of *H. pluvialis* were more susceptible than nonmotile cells to photooxidative stress and easy to die under the high light conditions [23,32]. In addition, our recent work indicated that nonmotile cells had a stronger astaxanthin accumulation capacity than motile cells [33]. During the encystment, the nonmotile cells have accumulated a certain amount of lipids and carbohydrates which play important roles in the synthesis of astaxanthin [10,15]. Once exposed to photooxidative stress, these accumulated lipids and carbohydrates can help nonmotile cells to rapidly synthesize and accumulate astaxanthin in a short time, and the increased astaxanthin further acts as ′sunscreen′ [34,35], protecting cells from photooxidative damage. In short, introducing an encystment stage between the vegetative growth stage and the red stage helps to improve the tolerance of vegetative cells to photooxidation, which can not only reduce the cell mortality rate of the red stage but also improve the astaxanthin production efficiency and stability. 

Another factor to consider that affects the production of astaxanthin is the IBD in the red stage. The cells in culture can shade each other to form the light-dark cycle, which affects the light exposure time individual cells receive. The higher the IBD, the more frequently the light-dark cycle, and the shorter the light exposure time individual cells receive [36]. At a certain light intensity, increasing the IBD can reduce the length of the cell’s light exposure time and decrease cell damage or death caused by photooxidation. Conversely, decreasing the IBD can increase cell light exposure time and the risk of cell damage and death from photooxidation, resulting in low biomass and astaxanthin productivity. According to our results, under the light intensity of 150 μmol photons m^−2^ s^−1^, the optimal IBD in the treatment group is 0.5, and the corresponding maximum astaxanthin content is 38.02 ± 2.40%, which was ca. 7.7% and 14.0% higher than that in 0.2 and 0.8 IBD group, indicating that optimizing the IBD in the red stage can further improve the production of astaxanthin. In addition, in many geographical locations, solar radiation can reach up to more than 2000 μmol photons m^−2^ s^−1^ that can cause cell death [36]. Shading strategies are widely used by producers during high solar radiation to reduce cell death occurred in the red stage. However, shading will not only affect the maximum utilization of potential useful solar energy, but also increase production costs. Compared with shading cultures, it is more economical to adjust appropriate IBD according to the intensity of solar radiation, and it is also more conducive to the maximum utilization of potentially useful solar energy. 

In summary, the work in this study provides a cost-effective method of *H. pluvialis* cultivation for the production of natural astaxanthin. Thereby, the motile cells are cultivated as inoculum under favorable conditions to produce more cells, then these cells encystment are induced to transform the nonmotile cells for enhancing the tolerance of cells to photooxidative stress, and finally, the nonmotile cells are adjusted to an optimal IBD and induced to accumulate astaxanthin in the red stage. This solved the problem of massive cell death occurring in the red stage and significantly improved biomass and astaxanthin production, which represents a good alternative to the traditional two-stage cultivation strategy of *H. pluvialis* for the production of astaxanthin.

## 4. Material and Methods

### 4.1. Strain and Culture Condition

*H. pluvialis* was obtained from the Center for Collections of Marine Algae (CCMA-451, GenBank accession number is MG847145) at Xiamen University (Xiamen, China). Stock cultures were maintained in BBM medium at 20 μmol photons m^−2^ s^−1^. 

Motile cells were cultivated in 3-L Erlenmeyer flasks containing 2.5-L 3N Bold Basal Medium (BBM) under continuous light (20 μmol photons m^−2^ s^−1^). 

The vegetative growth stage was carried out in BBM medium with an IBD of 0.2 (OD_680_) under 30 μmol photons m^−2^ s^−1^ of continuous light.

For the encystment stage, the 5-day-old vegetative cells were collected by centrifugation (2000 rpm, 2 min), then transferred into a fresh phosphorus-free BBM medium containing 1 g L^−1^ NaCl, and cultured at 30 μmol photons m^−2^ s^−1^ of continuous light for 3 days.

In the red stage, the cells at the end of the previous experiment (vegetative cells in the control group and green nonmotile cells in the treatment group) were collected, and then transferred into a fresh nitrogen-free and phosphorus-free medium to induce cells to synthesize astaxanthin. The experiments were carried out simultaneously in three groups of IBD 0.2, 0.5, and 0.8, and performed under 150 μmol photons m^−2^ s^−1^ of continuous light conditions for 8 days. 

All experiments were performed in triplicate in a 1-L glass column (inner diameter 5 cm; working volume 600 mL) at 25 ± 1 °C. Culture mixing was provided continuously by bubbling of filtered air enriched with 1.5% (*v/v*) CO_2_ at a flow rate of 100 mL min^−1^. The experiments were simultaneously repeated three times.

### 4.2. Analytical Methods

The algal biomass measured by a dry weight (DW) method [20]. Briefly, 10 mL cultured cells suspension were collected daily and filtered by passing through pre-weighed 1.2 μm Whatman GF/C filters (*m*_1_, g), then drying the cell mass at 90 °C for 500 mins to a constant weight (*m*_2_, g). The biomass was calculated with Equation (1):

(1) $$\mathrm{DW}\;\left({g\cdot L}^{-1}\right)=\left[m_{2}-m_{1}\right]\times 10^{3}/10$$

The cell mortality rate analyzed by cell numbers, which were counted by a Neubauer improved cell counting chamber and measured as cells·mL^−1^.

The astaxanthin concentration (AC) was determined photometrically [37]. A 10 mL cells were first treated with a solution of 5% (*w/v*) KOH in 30% (*v/v*) methanol at 75 °C for 10 min to destroy the chlorophyll. The treated pellet was extracted with 5 mL dimethyl sulfoxide (DMSO) and 25 μL acetic acid at 75 °C for 10 min. The extraction procedure was repeated several times until the pellet became colorless. Total astaxanthin was spectrophotometrically measured at 492 nm, and the astaxanthin concentration was calculated with Equation (2):

(2) $$\mathrm{AC}\left(\mathrm{mg}\cdot L^{-1}\right)=4.5\times A_{492}\times V_{a}\;\left(\mathrm{mL}\right)/10\;\left(\mathrm{mL}\right)$$

where *A*_492_ was the absorbance of extracts at 492 nm and *V*_a_ was the volume of extracts.

Astaxanthin content and astaxanthin content increase rate were calculated with Equation (3) and (4), respectively.

(3) $$C\;\left(\%\right)={\mathrm{AC}}_{t}\times 10^{-3}/{\mathrm{DW}}_{t}\times 100\%$$

(4) $$\mathrm{Astaxanthin}\;\mathrm{content}\;\mathrm{increase}\;\mathrm{rate}\;\left(\%\cdot {\mathrm{day}}^{-1}\right)=\left[C_{t\;}{-\;C}_{0}\right]/t$$

where *C*_t_ and *C*_0_ were the astaxanthin content on day t and day 0. 

IBDs were determined by the optical density method described in the previous study [38]. The cell morphology of different phases was observed using a Leica ICC50 W camera on Leica DM750 light microscope.

All experimental groups were simultaneously repeated three times, and the statistical analysis was performed by using mean, standard deviation, and maximum and minimum values.

## Figures and Tables

**Table 1:** The maximum value of biomass, biomass productivity, astaxanthin concentration, astaxanthin productivity, astaxanthin content, and astaxanthin content increase rate of the control- and treatment group under different IBDs.

Parameters	IBD: 0.2		IBD: 0.5		IBD: 0.8	
	Control Group	Treatment Group	Control Group	Treatment Group	Control Group	Treatment Group
Biomass (g L^−1^)	0.58 ± 0.06	0.92 ± 0.06	1.24 ± 0.13	2.02 ± 0.03	1.66 ± 0.08	2.62 ± 0.06
Biomass productivity (g L^−1^ d^−1^)	0.34 ± 0.04	0.44 ± 0.49	0.50 ± 0.08	0.66 ± 0.04	0.62 ± 0.04	0.72 ± 0.05
Astaxanthin concentration (mg L^−1^)	15.62 ± 1.35	32.26 ± 2.76	34.67 ± 2.91	72.51 ± 0.82	43.63 ± 3.03	87.35 ± 0.40
Astaxanthin productivity (mg L^−1^ d^−1^)	2.76 ± 0.33	5.42 ± 0.59	5.42 ± 0.05	11.36 ± 0.83	6.68 ± 0.66	12.37 ± 0.02
Astaxanthin content (mg g^−1^)	27.29 ± 0.67	35.29 ± 2.66	29.99 ± 1.43	38.02 ± 2.40	26.19 ± 0.59	33.35 ± 0.87
Astaxanthin content increase rate (mg g^-1^ d^−1^)	0.34 ± 0.01	0.54 ± 0.03	0.41 ± 0.10	0.74 ± 0.07	0.46 ± 0.06	0.65 ± 0.00
Cell mortality rate (*ca.*%)	25.85 ± 1.20	7.63 ± 3.35	23.14 ± 5.22	4.56 ± 0.27	19.08 ± 0.88	3.51 ± 2.48

The maximum value of each parameter was presented in the table. The cell mortality rate was obtained on day 8 of the red stage.
